# The clock drawing test as a screening tool for detecting cognitive decline: an analysis in adults and elderly people from Natal (RN)

**DOI:** 10.3389/fnhum.2026.1768390

**Published:** 2026-03-25

**Authors:** Letícia Ellen Cunha Pereira, Felipe Nalon Castro, Katie Moraes de Almondes

**Affiliations:** Postgraduate Program in Psychobiology, Universidade Federal do Rio Grande do Norte, Natal, Brazil

**Keywords:** clock drawing test (CDT), cognitive decline, early diagnosis, low education, neuropsychological assessment [205]

## Abstract

**Introduction:**

The Clock Drawing Test (CDT) is widely used as a screening instrument for cognitive decline due to its simplicity and rapid administration. Despite its widespread clinical use, evidence regarding its diagnostic performance in the Brazilian Northeast region remains scarce.

**Methods:**

This study examined CDT performance in a clinical sample of 113 adults and older adults assessed at a neuropsychology service in Northeastern Brazil, focusing on CDT ability to identify cognitive decline and the effects of age, educational level, and clinical diagnoses. The study tested the following hypotheses: (1) there is a significant association between age and CDT scores; (2) there are significant differences in CDT scores across different educational levels; (3) there are significant differences in CDT scores across different clinical conditions, indicating potential for differential diagnosis; and (4) the test would demonstrate high sensitivity, specificity, and accuracy in the overall sample, as well as high sensitivity in detecting each diagnostic condition. Using Shulman’s scoring method, descriptive analyses, Spearman’s correlation, Kruskal–Wallis tests and metrics of sensitivity, specificity, and accuracy were conducted.

**Results:**

The sample had a mean age of 65.19 years and was predominantly characterized by low educational attainment. A negative, albeit weak, correlation was observed between age and CDT scores, as well as significant differences across educational levels. Diagnostic group comparisons also revealed significant differences, most notably between cognitively unimpaired individuals and patients diagnosed with Major Neurocognitive Disorder due to Alzheimer’s disease. Although the CDT demonstrated adequate specificity, its overall sensitivity and accuracy were low. Sensitivity was high for Major Neurocognitive Disorder Due to Alzheimer’s Disease, moderate for Major Neurocognitive Disorder due to Non-Alzheimer’s Disease (Major Vascular Neurocognitive Disorder, Parkinson’s Disease, Mixed Dementia, Wernicke–Korsakoff syndrome and Major Frontotemporal Neurocognitive Disorder) and low for Mild Neurocognitive Disorder.

**Discussion:**

These findings demonstrate that Shulman’s method of CDT is not suitable for assessing cognitive decline in the illiterate and low-education population and raise important concerns regarding its standalone clinical utility, especially in specific neurological conditions. The present study underscores the need for future research employing alternative scoring methods and more representative samples to refine the applicability and diagnostic value of the CDT in clinical practice.

## 1 Introduction

Neurocognitive disorders represent a condition of increasing prevalence and a public health issue that demands ever greater attention and investigation from the international scientific community. It is estimated that, by 2030, brain diseases will account for half of the global economic burden associated with the treatment of non-communicable diseases (Knapp and Wong, 2020). By 2050, it is estimated that 152 million people will be living with dementia, resulting in 1.55 million deaths attributed to the disease (Nichols et al., 2022). Currently, most of these individuals are in low- and middle-income countries (Graeff et al., 2021). Even in this scenario, there is an underrepresentation of populations from the Global South, including countries in Latin America, in research (Moguilner et al., 2024). These countries contain vulnerable populations, more exposed to pollutants, structural inequalities, and sociopolitical factors associated with unhealthy aging (Hernandez et al., 2025). In this sense, socioeconomic disparities, high levels of air pollution, limited access to healthcare, increasing prevalence of communicable and non-communicable diseases, and low access to education are considered determinants of brain health in Latin American countries (Moguilner et al., 2024), leading to a prevalence of dementia of 8.5%, the highest among the continents (Prince et al., 2013). Thus, more accelerated brain aging was observed in these countries compared to Europe and Asia, demonstrating the adverse health effects caused by regional inequalities (Hernandez et al., 2025).

In Brazil, 1.8 million people aged 60 or older have been diagnosed with dementia, and projections indicate that 5.7 million people will be diagnosed by 2050 (Brasil, 2024). Within the country, there are also differences in representation among macro-regions, with most studies on cognition among the elderly being conducted in the Southeast (Castro-Costa et al., 2018). On the other hand, regions such as the Brazilian North, Northeast, and Midwest have lower economic development (5, 14, and 10% of GDP) (de Castro et al., 2020) and present significant socioeconomic and educational inequality. Therefore, lower education levels are the most important risk factor in these regions (de Castro et al., 2020). In the Northeast, factors associated with frailty were advanced age, presence of comorbidities, dependence in basic and instrumental activities, and negative self-perception of health status (Júnior et al., 2019). A study conducted in a city in the interior of Rio Grande do Norte, a state in the Brazilian Northeast, indicated that 65.9% of the sample of elderly people treated in the local health system presented cognitive impairment associated with age, illiteracy, depression, malnutrition or risk of malnutrition, dependence, and risk of falls (Pereira et al., 2020). In Natal, the capital of Rio Grande do Norte, frailty (unintentional weight loss, exhaustion, muscle weakness, slow gait, low level of physical activity) was attributed to 18% of the elderly sample, with a significant association between these symptoms and cognitive impairment (Medeiros, 2022). This information points to the need for further research exploring cognition and the early identification of diagnoses in these vulnerable regions, in order to expand intervention possibilities and create more effective public policies, especially in areas with less access to expensive biomarkers (Hernandez et al., 2025), such as the Brazilian Northeast.

Early diagnosis and intervention are crucial because advances in the neuroscience of aging have made it possible to characterize stages that precede the diagnosis of Major Neurocognitive Disorder, configuring cognitive decline as a continuous process. Currently, it is recognized that this process is influenced by modifiable risk factors, and not only by genetic determinants. Mitigating 14 key risk factors—including low education, hypertension, hearing loss, smoking, obesity, depression, sedentary lifestyle, diabetes, social isolation, excessive alcohol consumption, head injury, air pollution, high cholesterol and vision loss—can prevent or delay cases of cognitive decline (Livingston et al., 2024). In Brazil, almost 60% of dementia cases could be prevented by addressing 14 risk factors, primarily educational level, untreated vision loss, and middle-life depression (Suemoto et al., 2025). Thus, early diagnosis significantly increases the chances of implementing effective therapeutic interventions capable of slowing the progression to other conditions and potentially delaying its evolution to dementia, contributing to better health outcomes and improved quality of life for affected individuals (Graeff et al., 2021).

However, in clinical practice, cognitive changes are often detected only after spontaneous reporting of symptoms by patients or caregivers–which frequently occurs outside the ideal window for pharmacological or non-pharmacological interventions, such as lifestyle changes and psychosocial support. To mitigate this delay in diagnosis, a promising strategy is the systematic inclusion of routine cognitive assessment in asymptomatic older adults, especially in primary care (Jannati et al., 2024) by specialties such as general medicine, geriatrics, psychiatry, and neurology. Thus, neuropsychological screening tests play a central role in the early detection of possible cognitive decline (Kehl-Floberg et al., 2023). When combined with clinical observation and subjective reports from the patient and their family, these instruments increase the reliability of the diagnosis. Currently, the most widely used neuropsychological screening tools in clinical practice include the Montreal Cognitive Assessment (MoCA) (Nasreddine et al., 2005) and the Mini-Mental State Examination (MMSE) (Folstein et al., 1975), both widely validated and effective for assessing different cognitive domains. The Clock Drawing Test (CDT), in turn, is an efficient alternative, with quick application and good acceptability among the elderly, favoring its inclusion in routine screening contexts.

In 1986, Shulman proposed the use of the CDT as a tool for assessing elderly patients with possible cognitive disorders. Currently, the test is used to assess various cognitive domains, including visuospatial and visuoconstructive skills, graphomotor, and symbolic representation, attention, semantic memory, and executive function (Espinosa and Ribero, 2022). It is a cognitively complex task that requires the correct spatial representation of a circle, the sequential location of the 12 digits of the hours, and the appropriate insertion of the hands. Errors in performance may indicate deficits in semantic memory (object recognition), visuospatial skills, motor planning, and inhibition of perseverative responses (Handzlik et al., 2023). Due to its sensitivity to various cognitive impairments, the CDT is considered a useful tool in screening for conditions such as amnestic Mild Neurocognitive Disorder, Alzheimer’s Disease, Huntington’s Disease, Vascular Dementia, Parkinson’s Disease, Lewy Body Dementia (Rentz et al., 2021; Vergouw et al., 2018) and other neurodegenerative pathologies.

The Clock Drawing Test, as proposed by Goodglass and Kaplan, 1983, involves three steps: drawing the circumference of the clock, correctly positioning the 12 numbers, and adjusting the hands to the indicated time (11:10). Since then, several alternative assessment systems have been developed, with variations in terms of approach (quantitative, qualitative, or mixed) and scoring scale. According to Spenciere et al. (2017), the Shulman (from 0 to 5), Sunderland (from 0 to 10), and CDIS (from 0 to 20) systems have greater diagnostic accuracy. However, the literature still lacks consensus on which method would be most suitable for clinical and epidemiological use (Bougea et al., 2021).

The CDT is considered one of the most accessible screening tools for illiterate individuals or those with low levels of education, known for its ability to track the progression of cognitive decline by observing the progressive worsening of test performance, and to aid in the differential diagnosis between neurodegenerative diseases (Graeff et al., 2021). Studies indicate low sensitivity of the test for the detection of Mild Neurocognitive Disorder, showing greater relevance in the assessment of moderate and severe impairment (Esteves et al., 2022). Furthermore, the test has proven effective in identifying early cognitive changes in patients with Essential Tremor and Parkinson’s Disease, in whom initial cognitive dysfunction often presents as a dysexecutive syndrome (Bougea et al., 2021). Finally, studies indicate that the CDT has greater sensitivity than the MMSE in detecting and classifying cognitive decline, with a sensitivity of over 86% and a specificity greater than 96% with the Shulman method (Esteves et al., 2022), with reduced application time and feasibility of use by non-medical professionals (Jannati et al., 2024).

In Brazil, the test validation was carried out by the Martinelli Institute and its partners (Esteves et al., 2022). In a study conducted with octogenarians and nonagenarians from Siderópolis/SC (Schmidt et al., 2009) using the Sunderland method, the overall average on the CDT was 4.5, considered low. Studies conducted in Brazil also point to a significant difference when comparing the educational levels (Fabricio et al., 2014) and ages (Esteves et al., 2022) of the participants with the results on the instrument. It is worth noting that the aforementioned research was conducted in the Southern and Southeastern regions of Brazil, demonstrating the gap in research conducted in the context of Northeastern Brazil.

Given this, considering both the benefits of the Clock Drawing Test (CDT) as a screening tool and the need to improve its applicability in health services, as well as the lack of validated tools for assessing executive functions in elderly Brazilians (Esteves et al., 2022), the present study aimed to analyze the performance of adult and elderly individuals in the Clock Drawing Test and to verify the CDT ability to identify cognitive decline in a sample of a neuropsychology service in Natal (RN), exploring its potential across different age groups, educational levels, and diagnoses. A significant association was expected between age and CDT scores, as well as significant differences between educational levels and clinical presentations, indicating its potential in differential diagnosis. The test was also expected to demonstrate high sensitivity, specificity, and accuracy, proving its predictive power.

## 2 Materials and methods

### 2.1 Sampling procedure

The study adopted a cross-sectional design and was conducted with adult and elderly participants from Rio Grande do Norte and neighboring regions. The sample was obtained by convenience: the CDT was applied in the context of neuropsychological assessment due to suspected cognitive decline or prior indication for Deep Brain Stimulation (DBS) surgery, a procedure aimed at reducing motor symptoms in Parkinson’s Disease and which requires a neuropsychological report for continued treatment. Data collection took place over 10 years, between 2016 and 2025. Data were retrieved from digital medical records.

### 2.2 Participants

The initial sample contained 115 participants, but two results were excluded for not using the Shulman correction method, in order to standardize the analysis. The final sample consisted of 113 adult and elderly patients. The sample included healthy participants and patients with cognitive decline.

### 2.3 Procedure

The assessments were conducted at the Neuropsychology of Aging Service (SENE) of the Onofre Lopes University Hospital (HUOL) of Universidade Federal do Rio Grande do Norte (UFRN), in Natal (RN). Sessions were held weekly, totaling approximately 1 month for the completion of the evaluation. The assessments took place in a quiet and well-lit environment, ensuring adequate standardization of application conditions. The tests were conducted by psychology interns under the direct supervision of qualified psychologists. For application of The Clock Drawing Test (CDT), participants received a sheet with the outline of the pre-drawn clock face and were instructed to insert the numbers and hands, adjusting them to the time 11:10. CDT was applied as part of a broad neuropsychological battery, contributing to the assessment of executive and visuoconstructive functions. The tests used in the assessment included MoCA-Basic (Amatneeks and Hamdan, 2019), FAB (Beato et al., 2007), FDT (Sedó et al., 2015), RAVLT (de Paula et al., 2012) Digit Span (Nascimento, 2004), BPA (Rueda, 2013), Boston Naming Test (BNT) (Bertolucci et al., 2001), Stick Design Test (de Paula et al., 2013), Verbal Fluency Tasks (Esteves et al., 2015) and Corsi Block-Tapping Test (Corsi, 1972). The test results were interpreted based on their respective validations for the Brazilian populations, with a *Z*-score < −1.5 considered indicative of cognitive decline. The Functional Activities Questionnaire (FAQ) (Sanchez et al., 2011), Neuropsychiatric Inventory (NPI-Q) (Cummings et al., 1994) and Geriatric Depression Scale (GDS) (Castelo et al., 2009) were also used for assessing functionality, neuropsychiatric and depressive symptoms, respectively. Functional impairment is indicated when the FAQ score is greater than 4 (Smid et al., 2022). The clinical diagnoses were used as gold standard and obtained through the neuropsychological report delivered at the end of the assessment, which takes into account the results of the battery of tests, the patient’s and informant’s reports, clinical observation, imaging exams, and records made by the psychiatry and neurology teams, when available. The diagnosis follows the criteria of the DSM-5-TR: Major Neurocognitive Disorder is detectable through neuropsychological testing (*Z* < −2.0) in at least two cognitive domains, and presupposes cognitive complaint, cognitive impairment compared to previous state and functional impairment, not explainable by delirium or major psychiatric disorder (American Psychiatric Association, 2022). On the other hand, Mild Neurocognitive Disorder shows milder changes, also detectable through test results (*Z* < −1.5), with less functional impact, allowing the elderly person autonomy in basic, instrumental and advanced activities of daily living (American Psychiatric Association, 2022). Lastly, the diagnosis of Subjective Cognitive Decline (SCD) followed the criteria of The Subjective Cognitive Decline Initiative (Jessen et al., 2020): when cognitive complaints were present, but with normal performance on neuropsychological tests and maintained functionality. Diagnoses related to etiology (e.g., Alzheimer’s, Vascular Dementia) were also obtained from the DSM-5-TR criteria. Patients were considered healthy when no diagnosis had been assigned.

### 2.4 Ethical aspects

The study did not require approval from an Ethics Committee, in accordance with CNS Resolution 510/16, article 1, item V, which exempts research with databases without individual identification from evaluation by the CEP/CONEP system. The Free and Informed Consent Form (TCLE) was presented, in writing form or orally, considering illiterate individuals. The research was conducted in accordance with the guidelines of CNS Resolutions 466/12 and 510/16.

### 2.5 Measures and instruments

Shulman’s five-item scoring system was adopted in this research, being widely considered accurate for the diagnosis of dementia (Graeff et al., 2021). In this system, five points correspond to a perfect clock; four to discrete visuospatial errors; three to incorrect representation of the time, but with preserved visuospatial organization; two points indicate visuospatial disorganization of the numbers that prevents accurate time setting; one point indicates severe spatial disorganization; and zero points indicate a total inability to represent a clock. Scores below four points are indicative of cognitive decline (Graeff et al., 2021).

### 2.6 Statistical methods

Descriptive analyses were performed on the raw scores obtained in the CDT and the total number of patients with scores below the expected range, age, education level, sex, and clinical diagnosis, calculating frequencies or means and deviations. The normality of the scores distribution was tested using the Kolmogorov–Smirnov test, which indicated a lack of normality (*p* < 0.001) in the scores obtained in the CDT. Therefore, non-parametric tests were chosen. The Mann–Whitney U test was used to compare scores between sexes, while Spearman’s coefficient was adopted to compare scores obtained between different ages. A *ρ*-value between 0 and 0.49 was considered a weak correlation; from 0.50 to 0.69, a medium correlation; and from 0.70 to 1, a strong correlation (Ali, 2022). To analyze the formal education time data, the non-parametric Kruskal-Wallis test was used to compare the results obtained in each of the nine educational levels: illiterate, Incomplete Primary Education (IPE), Complete Primary Education (CPE), Incomplete High School (IHS), Complete High School (CHS), Complete Technical Education (CTE), Incomplete Higher Education (IHE), Complete Higher Education (CHE), and Postgraduate studies. Illiteracy is understood as the inability to read or write, and primary education comprises grades 1 through 9, while secondary education lasts 3 years. The Kruskal–Wallis test was also used to compare the main clinical diagnoses found after completion of the neuropsychological assessment. For the post-hoc analysis, the Dunntest with Bonferroni adjustment was used. Finally, sensitivity (probability of a positive result for cognitive decline in patients with clinical diagnosis, i.e., a true positive or positive result/total of patients with cognitive decline), specificity (probability of a negative result in healthy patients, i.e., true negative or negative result/total of patients without cognitive decline), and accuracy (probability of the test providing correct results, i.e., positive clinical patients + negative healthy/total sample) values were calculated for the cutoff point suggested in the literature (3/4, i.e., < 4 is indicative of decline), for the best overall cutoff point in this sample (the higher Youden Index, i.e., Sensitivity + Specificity − 1), and for each possible cutoff point of the test (0/1, 1/2, 2/3, 3/4 and 4/5), in addition to sensitivity valuesby diagnosis. Sensitivity, Specificity and Accuracy value of 50% equals chance level, and a < 50% value is worse than chance level. Results were considered significant when *p* ≤ 0.05. All analyses were conducted using R 4.5.2 software.

## 3 Results

The participants’ mean age was 65.19 years (±10.74), ranging from 42 to 91 years. Adult and elderly participants were kept in the same sample for the purpose of understanding the evolution of cognitive decline from middle age onwards, given that there is evidence of decline already in middle age (45–49 years) (Salthouse, 2019; Singh-Manoux et al., 2012), of more accelerated brain aging in Latin American countries from adulthood onwards due to cumulative exposure to the effects of socioeconomic inequalities (Moguilner et al., 2024; Hernandez et al., 2025), and greater recognition of early-onset dementia, before the age of 65 (Launer, 2019), as in many cases in this study. The sample comprised 59 women (52.2%) and 54 men (47.8%), with most participants being married (57.5%). Regarding educational level, 38% had not completed primary education, followed by participants with completed high school education (16%), completed higher education (11.5%), completed primary education (10.6%), illiterate individuals (8%), incomplete high school education (7.1%), incomplete higher education (5.3%), postgraduate education (1.7%), and completed technical education (0.9%). Educational data for one participant could not be retrieved and was therefore excluded from the Kruskal–Wallis analysis, resulting in a total of 112 observations. On the Clock Drawing Test (CDT), 64 participants (56.6%) scored below the cutoff point (3/4), suggesting possible cognitive decline, whereas 49 participants (43.4%) showed performance within the expected range.

The Mann–Whitney U test revealed a statistically significant difference between CDT scores obtained by female and male participants (*W* = 1,185; *p* < 0.02). The mean CDT score for the female group was 2.61 (±1.68), which was significantly lower than that of the male group, whose mean score was 3.37 (±1.54).

Regarding clinical diagnoses associated with cognitive decline identified during the neuropsychological assessment, cases of Mild Neurocognitive Disorder (33.6%), Major Neurocognitive Disorder due to Alzheimer’s disease (8.8%), Major Vascular Neurocognitive Disorder (5.3%), Major Neurocognitive Disorder due to Parkinson’s disease (4.4%), Mixed Dementia (2.7%), Wernicke–Korsakoff syndrome (2.7%), and Major Frontotemporal Neurocognitive Disorder (0.9%) were observed. Due to the low representation of the separate diagnoses, the diagnoses of Major Vascular Neurocognitive Disorder, Major Neurocognitive Disorder due to Parkinson’s disease, Mixed Dementia, Wernicke–Korsakoff syndrome and Major Frontotemporal Neurocognitive Disorder were combined into the Major Neurocognitive Disorder due to non-Alzheimer’s Disease subgroup (15.9%). Besides, 4.4% of the sample received a diagnosis of Subjective Cognitive Decline (SCD); however, since this nosological entity presupposes cognitive complaints with normal performance on neuropsychological tests, individuals with this diagnosis should obtain favorable results on the CDT. At the same time, in addition to subjective perception, there is a higher risk of progression to Mild Neurocognitive Disorder and Major Neurocognitive Disorder (6.7 and 2.3%, respectively) (Smid et al., 2022), making it difficult to classify this group as healthy or impaired. Therefore, the SCD group was excluded from sensitivity and ROC curve analyses (totaling 108 individuals) and comparisons between diagnostic groups. The remaining participants with cognitive impairment identified in the assessment received diagnoses of cognitive decline with inconclusive etiology (26.5%) and were therefore also excluded from the Kruskal–Wallis analysis to avoid distortions. Consequently, a total of 83 results were included in this analysis. Detailed sociodemographic characteristics are presented in Table 1.

**Table 1:** Sociodemographic characteristics.

Variables	Description									
Age (mean ± standard deviation)	65.19 (±10.74)	Min = 42	Max = 91							
Sex	Female	Male								
n	59	54								
%	52.2	47.8								
Marital status	Married	Widow(er)	Divorced	Single	Stable union	No data				
n	65	17	15	8	4	4				
%	57.5	15.0	13.3	7.1	3.5	3.5				
Education	Illiterate	IPE	CPE	IHS	CHS	CTE	IHE	CHE	Postgraduate	No data
n	9	43	12	8	18	1	6	13	2	1
%	8.0	38.0	10.6	7.1	16.0	0.9	5.3	11.5	1.7	0.9
Indicative of impairment based on CDT (cutoff point 3/4)	Yes	No								
n	64	49								
%	56.6	43.4								
Clinical diagnosis	Mild Neurocognitive Disorder	Major Neurocognitive Disorder Due to Alzheimer’s Disease	Major Neurocognitive Disorder due to non-Azlheimer’s Disease	Subjective Cognitive Decline	Healthy (no diagnosis)	Cognitive decline with inconclusive etiology				
n	38	10	18	5	12	30				
%	33.6	8.9	15.9	4.4	10.6	26.6				

IPE, Incomplete Primary Education; CPE, Complete Primary Education; IHS, Incomplete High School; CHS, Complete High School; CTE, Complete Technical Education; IHE, Incomplete Higher Education; CHE, Complete Higher Education.

**Figure 1:**
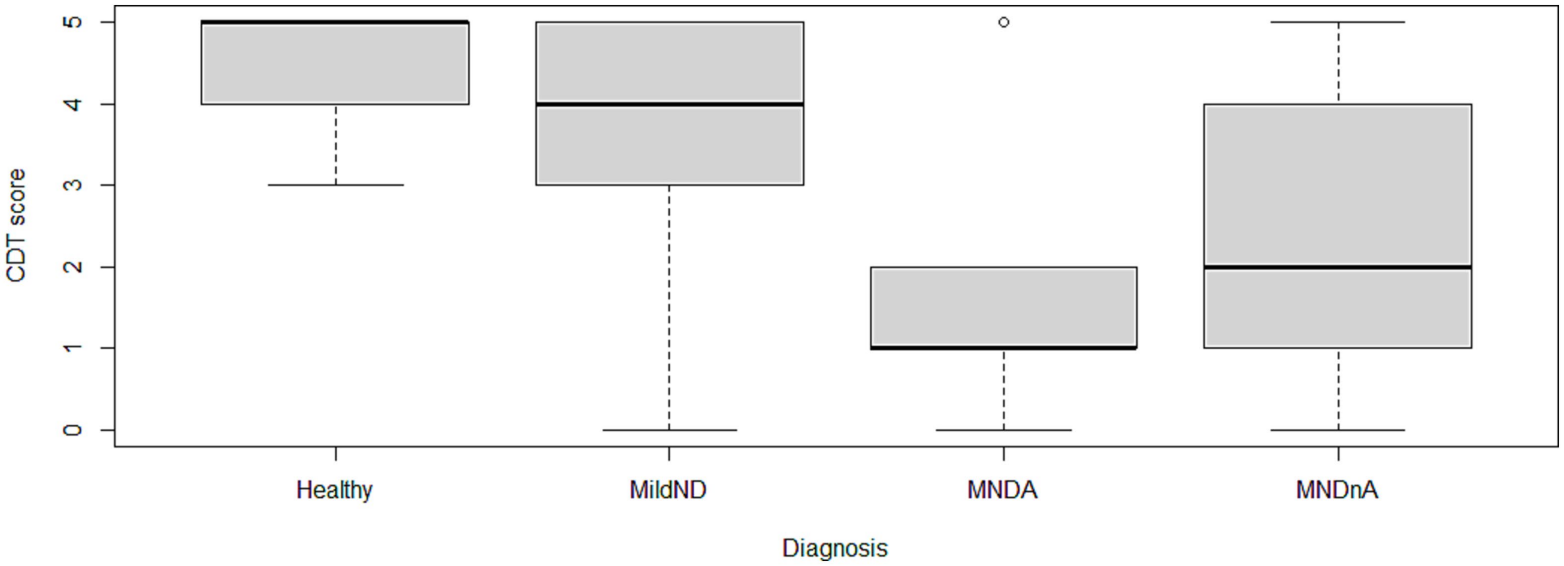
Boxplot: diagnosis × CDT score; MildND: mild neurocognitive disorder; MNDA: neurocognitive disorder due to Alzheimer’s disease; MNDnA: neurocognitive disorder due to non-Alzheimer’s disease.

The mean CDT score was 2.97 (±1.65) on a scale with a maximum score of 5. The score is considered low, taking into account the cutoff point of 3/4. Inferential analysis using Spearman’s correlation coefficient revealed a significant negative correlation between age and CDT performance (*ρ* = −0.26; *p* < 0.01), indicating that scores tended to be lower among older individuals; however, the magnitude of this association was weak. A summary of these correlations is presented in Figure 1. The Kruskal–Wallis test also indicated significant differences in CDT scores across educational groups (*p* < 0.001). However, *post hoc* analysis using the Dunn–Bonferroni test revealed statistically significant differences only between the following groups: illiterate individuals and those with completed higher education (*Z* = 3.57; *p* < 0.05), illiterate individuals and those with incomplete higher education (*Z* = −3.55; *p* < 0.02), individuals with incomplete primary education and those with completed higher education (*Z* = −4.23; *p* < 0.001), and individuals with incomplete primary education and those with incomplete higher education (*Z* = −3.82; *p* < 0.01). No other group comparisons were statistically significant.

The average scores among the diagnoses varied widely: the healthy group obtained the highest average score, 4.42 (±0.79), while the Major Neurocognitive Disorder Due to Alzheimer’s Disease group obtained the lowest, 1.50 (±1.35). The data are presented in Table 2. Similarly, comparison across clinical diagnostic groups indicated an overall significant difference (*p* < 0.001). The contrast between healthy participants and those diagnosed with Major Neurocognitive Disorder Due to Alzheimer’s Disease (*Z* = 3.968; *p* < 0.001); between Mild Neurocognitive Disorder and Major Neurocognitive Disorder due to Alzheimer’s Disease (*Z* = 3.036; *p* < 0.05); and between Healthy and Major Neurocognitive Disorder due to Non-Alzheimer’s Disease (*Z* = 3.209; *p* < 0.01) reached statistical significance. On the other hand, there was no significant difference between the groups Healthy and Mild Neurocognitive Impairment, Mild Neurocognitive Impairment and Major Neurocognitive Disorder due to non-Alzheimer Disease, and Major Neurocognitive Disorder due to Alzheimer Disease and Major Neurocognitive Disorder due to non-Alzheimer Disease. These findings are summarized in Table 3.

**Table 2:** Average scores by clinical diagnosis.

Clinical diagnosis	Mean (SD)
Mild neurocognitive disorder	3.42 (±1.57)
Major neurocognitive disorder due to Alzheimer’s disease	1.50 (±1.35)
Major neurocognitive disorder due to non-Alzheimer’s disease	2.44 (±1.54)
Healthy	4.42 (±0.79)

**Table 3:** Average score and inferential analysis.

Inferential analysis	Average score	
CDT score (mean ± SD)	2.97 (±1.66)*	
Correlation between age and CDT scores	*ρ* = −0.26**	*p* = 0.005
Comparison between educational levels and CDT scores	Kruskal–Wallis chi-squared = 37.171***	*p* < 0.001
Comparison between clinical diagnosis and CDT scores	Kruskal–Wallis chi-squared = 19.817***	p < 0.001
Comparison of scores between healthy—major neurocognitive disorder due to Alzheimer’s disease	*Z* = 3.968****	*p* < 0.001
Comparison of scores between mild neurocognitive disorder—major neurocognitive disorder due to Alzheimer’s disease	*Z* = 3.036****	*p* < 0.05
Comparison of scores between healthy—major neurocognitive disorder due to non-Alzheimer’s disease	*Z* = 3.209****	*p* < 0.01

*Arithmetic mean and standard deviation; **Spearman coefficient; ***Kruskal-Wallis; ****Dunn-Bonferroni *post-hoc*.

**Figure 2:**
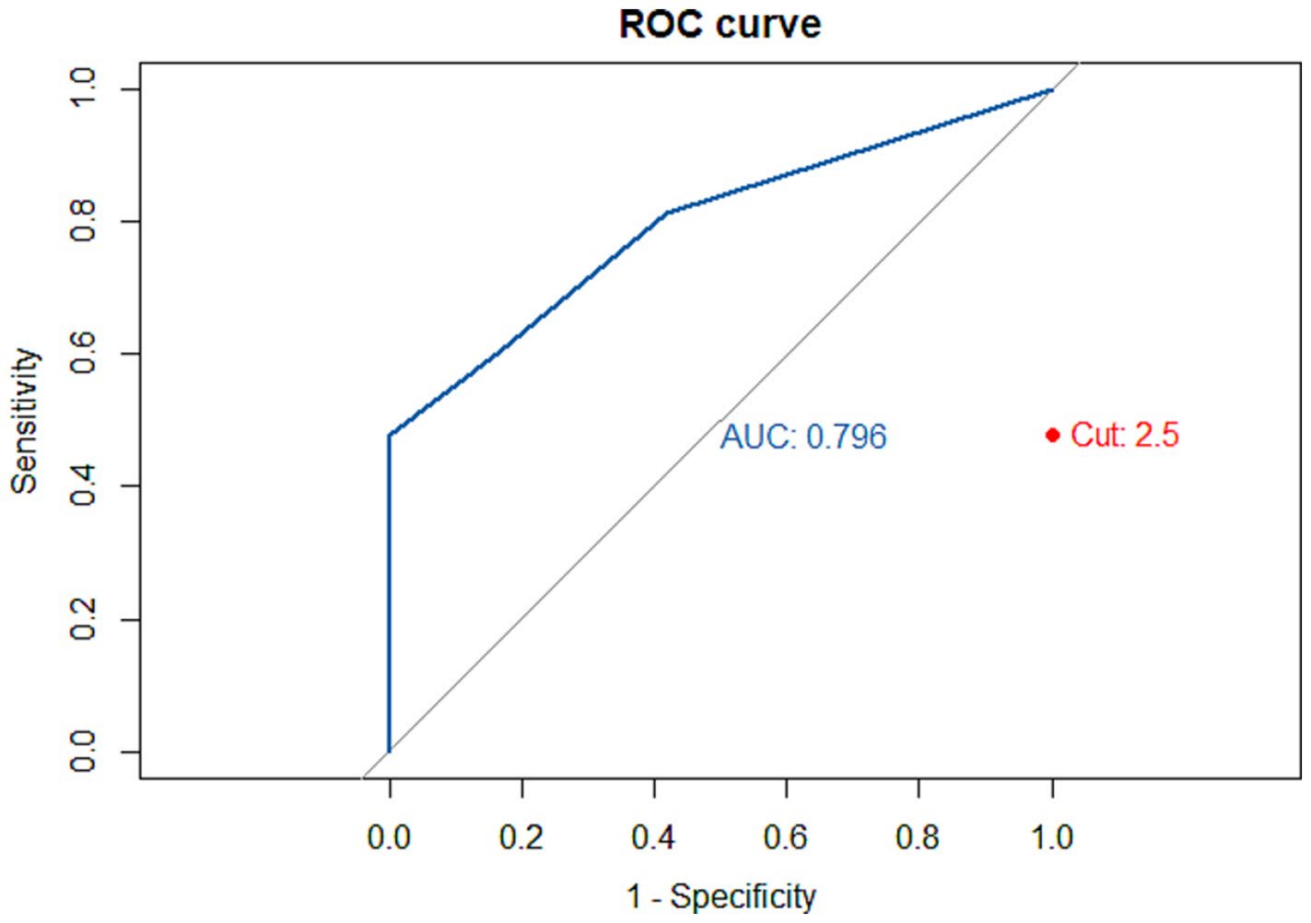
ROC curve.

Subsequently, the sensitivity, specificity, and accuracy of the Clock Drawing Test (CDT) were calculated. For the cutoff score of 3/4, specificity was 83.3%, which is considered high. However, sensitivity was 60.4%, and accuracy was 63.0%, both of which were relatively low. The optimal cutoff point for the sample was 2/3 (i.e., < 3 is indicative of cognitive decline), yielding a specificity of 100%, but sensitivity of 47.9% and accuracy of 53.7%, which also remained very low, worse and close to chance level, respectively. Even when the cutoff point was the maximum (4/5), meaning that only a score of 5 would indicate an absence of decline and at which high sensitivity would be expected, the value was 81.3%, showing that about 18.7% of patients with cognitive decline were able to score 5 on the CDT. These data are presented as a receiver operating characteristic (ROC) curve in Figure 2 and Table 4. It is important to highlight that although Shulman’s scoring system includes six possible values (0–5), the ROC analysis correctly generated seven thresholds and seven distinct sensitivity–specificity pairs. The apparent limited number of inflection points in the figure reflects the discrete ordinal nature of the scale and the vertical clustering of points when specificity equals 1.0.

**Table 4:** Sensitivity/specificity by diagnostic group (cutoff point 3/4).

Cutoff point	Sensitivity	Specificity	Accuracy
0/1	8.3%	100%	18.5%
1/2	29.1%	100%	37.0%
2/3	47.9%	100%	53.7%
3/4	60.4%	83.3%	63.0%
4/5	81.3%	58.3%	78.7%

Finally, regarding sensitivity by diagnostic group, the results were not considered reliable due to the high variability observed within the 95% confidence intervals. Nevertheless, among participants without cognitive decline, correct identification occurred in 83.3% of cases, a high value as already mentioned before. In addition, the CDT accurately identified 90% of cases associated with major neurocognitive disorder due to Alzheimer’s disease and 72.2% of Major Neurocognitive Disorder due to Non-Alzheimer’s Disease, considered high and moderate, respectively. On the other hand, CDT identified 44.7% of cases of Mild Neurocognitive Disorder, a low value and worse than chance level. The complete results are presented in Table 5.

**Table 5:** Sensitivity/specificity by diagnostic group (cutoff point 3/4).

Clinical diagnosis	Sensitivity	Confidence intervals 95%
Mild neurocognitive disorder	44.7%	0.286–0.617
Major neurocognitive disorder due to Alzheimer’s disease	90%	0.555–0.997
Major neurocognitive disorder due to non-Alzheimer’s disease	72.2%	0.465–0.903
Healthy (specificity)	83.3%	0.515–0.979

## 4 Discussion

This study aimed to analyze the performance of adults and older adults on the Clock Drawing Test (CDT), assessing its potential for detecting cognitive decline while considering different age groups, levels of education, and clinical diagnoses. A key strength of this study is its focus on cognitive aging within the Northeastern region of Brazil, thereby departing from the predominance of research conducted in the Global North (Long et al., 2023) and expanding the focus to marginalized populations, in which adverse environments affect dendritic branching and synapse formation (Moguilner et al., 2024). This context of vulnerability is illustrated by the high proportion of participants who did not complete primary education (38.0%), contrasted with the very small proportion who completed postgraduate education (1.7%). Thus, the sample closely reflects the reality of the Brazilian older adult population, which is characterized by low levels of formal education (Esteves et al., 2022).

With respect to sociodemographic data, the predominance of women (52.2%) seeking services for the investigation of cognitive decline supports the understanding of female sex as a risk factor for the development of most neurocognitive disorders. This association may be explained, in part, by hormonal changes related to menopause (Hogervorst et al., 2023), associated with reduced brain volume and increased beta-amyloid deposition (Moguilner et al., 2024). On the other hand, these factors interact with environmental factors and gender disparities, since Brazilian women face disadvantages due to their caregiving role, performance of domestic activities, low economic independence, restricted social life, and a higher prevalence of chronic diseases (Júnior et al., 2019). Thus, gender inequality at the national level is also associated with differences in cortical thickness (Moguilner et al., 2024). Accordingly, women showed significantly lower mean CDT scores, corroborating previous findings. However, these results are strongly influenced by the fact that women generally seek healthcare services more frequently, and therefore should be interpreted with caution. Conversely, most participants were married (57.5%), a characteristic not explored in depth in this study, but considered protective against dementia due to its association with lower depressive symptoms and psychiatric distress (Correro et al., 2023).

In addition to sex, age is also recognized as a predisposing factor for cognitive decline, with aging representing the primary risk factor for most dementias (Jia et al., 2020). In this regard, the present analysis corroborated previous studies (Esteves et al., 2022; Graeff et al., 2021) demonstrating poorer CDT performance with increasing age. However, the observed correlation was weak (*ρ* = −0.26), likely due to the limited variability in scores, which ranged from 0 to 5 under the scoring method employed. Furthermore, another important risk factor for cognitive decline is fewer years of formal education (Long et al., 2023). Higher levels of education are associated with enhanced brain development, including increased dendritic growth and cerebral circulation, which contribute to better performance on neuropsychological tests (Espinosa and Ribero, 2022). Consequently, cognitive impairment is more prevalent among individuals with lower educational attainment, with education being the most important factor related to cognitive function among Brazilians (Bertola et al., 2021). Therefore, the present findings support this premise and are consistent with previous studies (Fabricio et al., 2014; Graeff et al., 2021). The results highlight the importance of early childhood education for vulnerable populations in Brazil and the need for public policies to reduce illiteracy and school dropout rates (Suemoto et al., 2023).

Regarding differential diagnosis, the study sought to determine whether clinical diagnoses established at the end of the neuropsychological assessment were associated with significantly different CDT scores. The analysis indicated an overall significant difference among diagnostic groups; however, pairwise comparisons revealed a reliable difference only between healthy participants and those diagnosed with Major Neurocognitive Disorder Due to Alzheimer’s Disease, Mild Neurocognitive Disorder and Major Neurocognitive Disorder due to Alzheimer’s Disease, and healthy participants and Major Neurocognitive Disorder due to Non-Alzheimer’s Disease. Thus, the findings regarding differences when compared to healthy groups reinforce the potential utility of the CDT for detecting cognitive decline associated with Alzheimer’s disease and other neurodegenerative pathologies, like Vascular Dementia (Rentz et al., 2021). Also demonstrate that patients with Mild Neurocognitive Disorder did not present significantly different patterns from healthy patients, showing a weakness of the CDT in detecting subtle changes, as the literature already suggested (Esteves et al., 2022). On the other hand, the significant difference between Mild Neurocognitive Disorder and Major Neurocognitive Disorder due to Alzheimer’s Disease suggests that CDT is able to capture the progression of cognitive decline within the neurodegeneration continuum, as already suggested (Graeff et al., 2021). Moreover, the absence of additional significant differences supports the notion that distinctions among neurocognitive disorders may be better captured through qualitative analysis of the drawing process and error patterns, rather than solely by the final product or total score (Souillard-Mandar et al., 2015).

Beyond diagnostic comparisons, the study evaluated whether the CDT accurately identified the presence or absence of cognitive decline relative to diagnoses obtained through comprehensive neuropsychological assessment. Using a cutoff score of 3/4, the CDT correctly identified normal cognition in 83.3% of participants without a diagnosis, confirming its high specificity and ability to detect cognitively healthy individuals. However, sensitivity and accuracy were low, indicating that individuals with cognitive decline were not consistently identified by the CDT using the Shulman’s scoring, contrary to what was suggested (Esteves et al., 2022). Thus, even though the sample originates from a region with a high prevalence of risk factors at the socioeconomic level (Medeiros, 2022; Pereira et al., 2020) and is mostly composed of patients with low levels of education, many patients with cognitive decline obtained the maximum score on the CDT using the Shulman correction method. Therefore, even if the test is considered accessible to the illiterate and low-education population (Graeff et al., 2021), this suggests that the instrument performs no better than chance in detecting cognitive impairment when used in isolation in these groups.

Furthermore, the CDT demonstrated high sensitivity for cases of major neurocognitive disorder due to Alzheimer’s disease and low sensitivity for mild neurocognitive disorder, corroborating previous findings (Esteves et al., 2022; Rentz et al., 2021). It also correctly identified a moderate value of cases of Major Neurocognitive Disorder due to Non-Alzheimer’s Disease, demonstrating its potential use in cases beyond Alzheimer’s Disease, as already pointed out. In that regard, the test demonstrated detection potential for observed decline in Major Neurocognitive Disorder Due to Alzheimer’s Disease and Major Neurocognitive Disorder due to Non-Alzheimer’s Disease (Vascular Dementia, Parkinson’s Disease, Wernicke-Korsakoff Syndrome, Frontotemporal Dementia, and Mixed Dementia), but not for Mild Neurocognitive Disorder.

In summary, using Shulman’s method, higher age and lower education level were associated with the worst and best performance on the CDT, as already pointed out in the literature. On the other hand, patients with cognitive decline confirmed by comprehensive neuropsychological assessment, even with low education levels and belonging to a vulnerable region, obtained high scores and were not significantly detected by the test, even with high cut-off points. These results indicate that Shulman’s correction method is not suitable for cognitive assessment in patients from vulnerable regions with low levels of education. Even so, the test has the potential to detect some neurodegenerative conditions, but is not effective in detecting Mild Neurocognitive Disorder.

## 5 Conclusion

This study provided insight into data obtained from a neuropsychology service in Natal, Rio Grande do Norte, in northeastern Brazil, offering a detailed perspective on the cognitive performance of adults and older adults on a neuropsychological screening test. The main strength of the research conducted lies in the analysis of CDT results in a sample that is underrepresented in research, revealing the cognitive performance of patients and enabling the application of the data in similar contexts. The results obtained are applicable to the assessment of middle-aged and elderly adults. There was a significant association between age and CDT scores, as well as significant differences in scores across age groups and clinical conditions, as expected. However, the test did not demonstrate high accuracy or sensitivity in detecting overall cognitive decline in the overall sample and in Mild Neurocognitive Disorder, indicating that the correction method is not suitable for application in populations similar to the one analyzed.

This study had some limitations and its results should be considered carefully. The primary limitation was the use of convenience data derived from broader neuropsychological assessments. As a result, a relatively small sample was used, limiting the generalizability of the results, and potential confounding variables, such as psychiatric disorders and sensory impairments, could not be controlled. Additionally, the inclusion of participants with diverse conditions and varying prevalence rates hindered the formation of balanced groups of education and clinical diagnosis. The Mild Neurocognitive Disorder group also could not be separated by etiologies or affected cognitive domains, which may also have been a cause of its low sensitivity. Furthermore, the use of a cross-sectional methodology does not allow for the determination of causality, which requires longitudinal and experimental research with similar topics and populations. The extended time frame over which assessments were conducted (10 years) may also have introduced temporal and generation bias due to cohort differences, which may have significantly affected the results obtained. On the other hand, the application, even over such a long period, used the same tests and application protocols, reducing measurement error. Furthermore, even though the assessment utilized a variety of neuropsychological tests, imaging exams, and multiple reports, it is necessary to highlight the potential bias from incorporating the CDT into the assessment to determine cognitive decline. Another relevant limitation concerns the use of a scoring method with limited score variability, underscoring the importance of future studies employing alternative, well-established scoring systems in vulnerable populations. It is also imperative to investigate how CDT performance evolves alongside the progression of the neurodegenerative conditions examined. Overall, the findings are expected to contribute to the scientific literature and to clinical practice in neuropsychology with adults and older adults.

## Data Availability

The raw data supporting the conclusions of this article will be made available by the authors, without undue reservation.
